# Seasonal variations in vertical migration of glacier lanternfish, *Benthosema glaciale*

**DOI:** 10.1007/s00227-012-1953-2

**Published:** 2012-06-05

**Authors:** Eivind Dypvik, Anders Røstad, Stein Kaartvedt

**Affiliations:** Red Sea Research Center, 4700 King Abdullah University of Science and Technology, Thuwal, 23955-6900 Saudi Arabia

## Abstract

The seasonal variations in glacier lanternfish (*Benthosema glaciale*) vertical distribution and diel vertical migration (DVM) were studied by use of a bottom-mounted upward-facing 38 kHz echo sounder deployed at 392 m depth and cabled to shore in Masfjorden (~60°52′N, ~5°24′E), Norway. Acoustic data from July 2007–October 2008 were analyzed, and scattering layers below ~220 m during daytime were attributed to glacier lanternfish based on net sampling in this, and previous studies, as well as from analysis of the acoustic data. At these depths, three different diel behavioral strategies were apparent: normal diel vertical migration (NDVM), inverse DVM (IDVM), and no DVM (NoDVM). NoDVM was present all year, while IDVM was present in autumn and winter, and NDVM was present during spring and summer. The seasonal differences in DVM behavior seem to correlate with previously established seasonal distribution of prey. We hypothesize that in regions with seasonally migrating zooplankton, such as where calanoid copepods overwinter at depth, similar plasticity in DVM behavior might occur in other populations of lanternfishes.

## Introduction

Myctophids (Myctophidae) are mesopelagic fishes distributed in all the world’s oceans (Dalpadado and Gjøsæter 1988; Cherel et al. 2010), playing an important role in the marine ecosystems (Gjøsæter and Kawaguchi 1980; Cherel et al. 2010) as trophic links between zooplankton (Kinzer and Schulz 1985; Shreeve et al. 2009) and piscivorous predators (Giske et al. 1990; Markaida and Sosa-Nishizaki 2003; Doksæter et al. 2008; Hedd et al. 2009). They are also likely to play a major role in the vertical flux of organic matter from the upper productive layer to deeper layers (Radchenko 2007; Hernandez-Leon et al. 2010). Their vertical distribution and diel vertical migration behavior affect such interactions and processes, making it important to reveal these patterns. The myctophid glacier lanternfish (*Benthosema glaciale*) and the sternoptychidae pearlside (*Maurolicus muelleri*) are the dominant mesopelagic fish in Norwegian fjords (Aksnes et al. 2004; Kristoffersen and Salvanes 2009). In Masfjorden, pearlside dominates acoustic scattering layers (SL’s) shallower than 200 m, while glacier lanternfish dominates the SL’s below 200–250 m (Giske et al. 1990; Bagøien et al. 2001; Kaartvedt et al. 2009; Dypvik et al. 2012).

Diel vertical migration (DVM) is commonly referred to as a trade-off between feeding opportunities and predation risk induced by changes in light intensity (Clark and Levy 1988; Pearre 2003; Cohen and Forward 2009; Ringelberg 2010). Thus, light is the proximate cause of DVM influencing the time of migration and vertical extent of migration (Ringelberg and Van Gool 2003; Staby and Aksnes 2011), while the distribution of food and predation risk are regarded as major drivers for fish vertical distribution and migration (Clark and Levy 1988; Bailey 1989; Neilson and Perry 1990; Sutton and Hopkins 1996). However, size (Busch and Mehner 2012), internal factors such as energy reserves (Hays et al. 2001) and hunger (Pearre 2003), and external factors such as currents (Bennett et al. 2002) and temperature (Wurtsbaugh and Neverman 1988; Sogard and Olla 1996; Mehner et al. 2010) may also influence vertical distribution and migration. In normal DVM (NDVM), individuals ascend toward the surface at night before descending to deeper waters during the day. In the less common inverse DVM (IDVM), individuals ascend in the water column at daytime and descend during the night (Pearre 2003).

Glacier lanternfish is known to carry out NDVM (Halliday 1970; Roe and Badcock 1984; Sameoto 1988), IDVM (Kaartvedt et al. 2009; Dypvik et al. 2012) or display no diel vertical migration (NoDVM) (Roe and Badcock 1984; Albikovskaya 1988; Kaartvedt et al. 2009). The depth distribution of myctophids is size dependent with larger fish distributed deeper than smaller individuals (Willis and Pearcy 1980; Gartner et al. 1987; Dypvik et al. 2012). Therefore, differences in conspicuousness (Hays et al. 1994), vision (capabilities) for detecting prey (Warrant and Locket 2004), internal state (satiation and hunger) (Cailliet and Ebeling 1990; Staby et al. 2011), and motivation (Rosland and Giske 1997; Busch and Mehner 2012) are expected to result in a mixture of migration patterns, which may occur simultaneously.

Glacier lanternfish feeds on a variety of zooplankton (Gjøsæter 1973; Roe and Badcock 1984; Sameoto 1988), but seems to prefer calanoid copepods, especially *Calanus* (Sameoto 1988, 1989; Baliño and Aksnes 1993; Dypvik et al. 2012). The main pattern in seasonal zooplankton abundance and vertical distribution at the site for this study is established from previous studies at the same or adjacent locations. In spring and summer, most zooplankton are distributed in the upper part (<30 m) of the water column (Rasmussen and Giske 1994). However, during autumn and winter, the highest biomass of zooplankton is below 150 m (Giske et al. 1990; Baliño and Aksnes 1993; Bagøien et al. 2001). This is because zooplankton vanishes from upper layers as primary production declines, but also because of seasonal vertical migration among the main calanoid copepod species, *Calanus* spp., which leaves upper waters, descending for “overwintering” in mid-waters (Bagøien et al. 2001). In Norwegian waters, this seasonal descent may begin in summer (Kaartvedt 2000). Myctophids are capable of feeding at mesopelagic depths (Roe and Badcock 1984; Sameoto 1988; Pusch et al. 2004), so that the seasonal migration of *Calanus* to deep waters may represent an important food source in the daytime habitat of the fish (Dypvik et al. 2012). In Norwegian fjords, glacier lanternfish exercise a strong predation pressure on overwintering *Calanus* (Bagøien et al. 2001) and can influence their vertical distribution (Kaartvedt 1996).

Mesopelagic fish can be studied by use of echo sounders as they tend to aggregate into SL’s (Holton 1969; Godø et al. 2009; Kaartvedt et al. 2009). Normally, acoustic studies of mesopelagic fish are carried out in periods restricted by time or seasonality (Collins et al. 2008; Godø et al. 2009; Kloser et al. 2009), and to our knowledge, there is no systematic study addressing how migration patterns in a population of glacier lanternfish, or other myctophids, may vary throughout a year. However, the use of moored echo sounders can give long-time acoustic data series (Brierley et al. 2006; Doksæter et al. 2009; Staby et al. 2011). Here we take advantage of the unique opportunity for a long-term study offered by a deep fjord where populations of mesopelagic fish are easily accessible. We present data from 16 months of continuous acoustic registrations (July 2007–October 2008), enabling us to address the seasonal patterns of diel vertical behavior, unveiling the relative occurrence and consistency of NDVM, IDVM, and NoDVM by glacier lanternfish.

At the outset of this study, we hypothesized that the patterns of glacier lanternfish DVM would vary seasonally, as recently documented for the pearlside (Staby et al. 2011), likely in relation to the seasonal distribution of prey. Given the general seasonal zooplankton dynamics of Norwegian fjords, we hypothesize that during spring and summer, when the abundance of potential prey is high in near-surface waters, the glacier lanternfish migrate to the upper part of the water column at night (NDVM) to feed, avoiding visual predators in the bright surface waters during daytime. Conversely, we hypothesize that during autumn and winter, when food is sparse in upper waters, and at seasonal high in mid-waters, as seasonally migrating copepods have descended to their overwintering habitat, glacier lanternfish restrain from migrations to the surface at night, rather exploiting the prey in mid-waters by performing IDVM toward favored light conditions in shallower waters during daytime (Dypvik et al. 2012). Furthermore, for the NoDVM layer, we investigate two alternative hypotheses: (1) the population really does split into migrating and non-migrating components (Pearcy et al. 1979) or (2) migrations are undertaken also among the NoDVM component, but not detected by standard methods because they are asynchronous, so that a proportion of the population always is present in deep waters (Sutton and Hopkins 1996; Pearre 2003). Since our approach facilitates studies of individuals, we have the possibility of addressing these competing hypotheses.

## Materials and methods

**Fig. 1:**
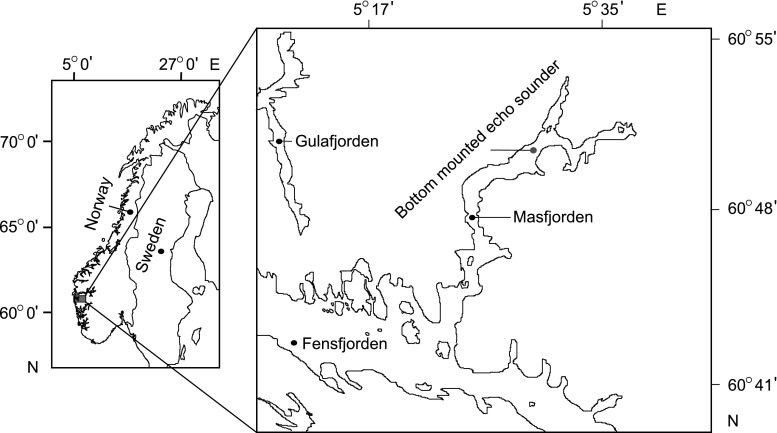
Map depicting location of the bottom-mounted echo sounder (*gray circle*) at 392 m depth in Masfjorden, Norway

The study was undertaken in Masfjorden (~60°52′N, ~5°24′E), Norway (Fig. 1). For a detailed description of the fjord, see Kaartvedt et al. (1988), Baliño and Aksnes (1993). The deeper parts of the water column (~200–390 m) are particularly in focus as glacier lanternfish dominates the acoustic backscattering, and IDVM and NoDVM occur in this interval (Dypvik et al. 2012).

A mooring with an upward-facing 38 kHz Simrad EK60 split-beam echo sounder (7.1° beam width), cabled to shore for power and transmission of data, was deployed at 392 m in July 2007 and retrieved in October 2008. Detailed descriptions of the echo sounder system and parameter settings are given in Kaartvedt et al. (2009). Data were recorded continuously, apart for intermittent periods following power failures, giving a total of 218 full days of records spanning all seasons. A minimum number of 4 days per month were included in this study (i.e. Sept. 2007 and Oct. 2008). We classify September–November as autumn, December–February as winter, March–May as spring, and June–August as summer.

The results are presented as average echograms illustrating the average *S*_v_ (dB) values for the entire water column (~0–390 m) for each month, and in greater detail from selected months representing each season for the section ~200–390 m, which is of particular interest for this study (called monthly echograms). *S*_v_ values are logarithmic measurements of the volume acoustic backscattering or accumulated TS (target strength), TS representing the echo of a single individual (MacLennan and Simmonds 1992). The seasonal patterns of the vertical distribution and migration of glacier lanternfish is assessed by studying the scattering layers in monthly echograms. These monthly echograms were made in Matlab by dividing each day into 30 s intervals, and for each time interval average all the pings for the given month. In this way, all the acoustic records from the entire registration period are included in the presentations. In addition, to better illustrate any activity in the NoDVM layer, which by definition was expected to have little vertical migration, we randomly picked 1 day for each season (18 October 2007; 2 February; 12 March 2008; 8 August 2008), focusing on the 300–390 m depth interval. For these days, we furthermore selectively chose examples where vertical swimming was seen in the compressed, daily echograms, and portrayed these at higher resolution for better depicting individual behavior.

Abundance estimates to identify the variation in concentration of glacier lanternfish in different depth intervals between day and night were made by echo integration. This was done with day and depth divided into blocks of 1 h and 10 m respectively between ~250 and 390 m for days where 24 h of echo data were successfully retrieved (from min. 4 days in Sept. 2007 and Oct. 2008, to max. 24 days in May 2008). Time for sunrise and sunset was set to the 15th of each corresponding month, as an approximate for each month, determining time allocated to day and night. Glacier lanternfish makes up the main part of the volume backscattering (*S*_v_) at −90 dB, while larger fish results in *S*_v_ values greater than −64 dB (Bagøien et al. 2001). Thus, values retrieved by echo integration at −64 dB were subtracted from the echo integration at −90 dB to exclude fish larger than glacier lanternfish (Bagøien et al. 2001). The concentrations (individuals m^−3^) were derived from measurements of *S*_v_ and target strength (TS, see description below) by dividing the linear *S*_v_ values with the linear TS (MacLennan and Simmonds 1992).

TS depends on the size, anatomy, and behavior of the organism, as well as the frequency of the echo sounder (MacLennan and Simmonds 1992). For precise estimation of concentration, the TS of glacier lanternfish was obtained monthly by means of automatic target tracking (TT), performing TT for 3 days of every month within a range of 10–50 m from the transducer (~340–380 m depth). This limited range was chosen as the resolution of acoustic data decreases with distance from the transducer, and so does the precision of the TS estimates. Minimum track length was set at 10 ping, maximum ping gap to 1 ping and gating range to 0.3 m during tracking. TS values between −65 and −50 dB were regarded as glacier lanternfish, as TS values stronger than −50 dB indicate fish of greater size (Foote 1980; MacLennan and Simmonds 1992). A minimum value of −65 dB was chosen in accordance with a previous acoustic study of glacier lanternfish (Torgersen and Kaartvedt 2001). Tracks of individual fish obtained by TT were also used for analysis of swimming behavior. Vertical swimming speeds of individuals were analyzed together with visual inspection of daily 24 h echograms in order to investigate any asynchronicity in the DVM pattern of the deeper living part of the population.

Both TT and echo integration were performed in the post processing program Sonar_5 pro version 5.9.9 (Balk and Lindem 2007).

The migration patterns below 250 m as assessed from the monthly echograms were compared with changes in concentration as assessed based on combining the measurements of *S*_v_ and TS. The maximum concentration of glacier lanternfish estimated below 250 m each day was assumed to reflect the total population below 250 m. Increase and/or decrease in concentration, before and after the time of migration of a specific layer of glacier lanternfish, was used as a proxy for the relative proportion of glacier lanternfish with NoDVM, IDVM, and NDVM. The speed of migration by individuals in SL’s was calculated by analyzing the distance of descent/ascent over time in SL’s.

The continuous acoustic measurements were complemented by sampling during research cruises 1–4 November 2007 with R/V “Haakon Mosby” (University of Bergen, IMR) and 3–7 October 2008 with R/V “Trygve Braarud” (University of Oslo). Results from the physical oceanography, zooplankton, and trawling studies from these field campaigns are used in the interpretations, but are presented elsewhere (Staby et al. 2011; Dypvik et al. 2012).

The behavioral patterns described in this study can with confidence be ascribed to glacier lanternfish. Previous studies covering spring and summer (Rasmussen and Giske 1994; Kaartvedt et al. unpublished), autumn (Bagøien et al. 2001; Kaartvedt et al. 2009), winter (Giske et al. 1990; Baliño and Aksnes 1993; Bagøien et al. 2001), and trawl data obtained during the present study (Staby et al. 2011; Dypvik et al. 2012), as well as subsequent studies (Kaartvedt et al. unpublished) have shown that glacier lanternfish are the main cause of backscattering deeper than 250 m in Masfjorden. Catches at these depths consist of specimens >4.5 cm, with a prevalence of individuals >6 cm below 300 m (Kaartvedt et al. 2009; Dypvik et al. 2012), that is adult individuals (Gjøsæter 1981). Pelagic shrimps are also common in the deep waters of Masfjorden (Kaartvedt et al. 1988, 2009) and may contribute somewhat to the backscatter. However, the SL’s below 200 m in Masfjorden are pronounced also at 18 kHz (Kaartvedt et al. 2008), signifying that the backscatter mainly consists of mesopelagic fish (Torgersen and Kaartvedt 2001; Love et al. 2004; Godø et al. 2009; Kloser et al. 2009). Furthermore, the average TS of the targets studied here (see “Results”; “Target strength and concentration estimates in deep water”) is in accordance with previous studies of lanternfishes (Torgersen and Kaartvedt 2001; Yasuma et al. 2003; Kaartvedt et al. 2009), while shrimps have a TS about one magnitude weaker than that of glacier lanternfish (Benoit-Bird and Au 2001; Torgersen and Kaartvedt 2001).

## Results

### Main patterns in acoustic backscatter

**Fig. 2:**
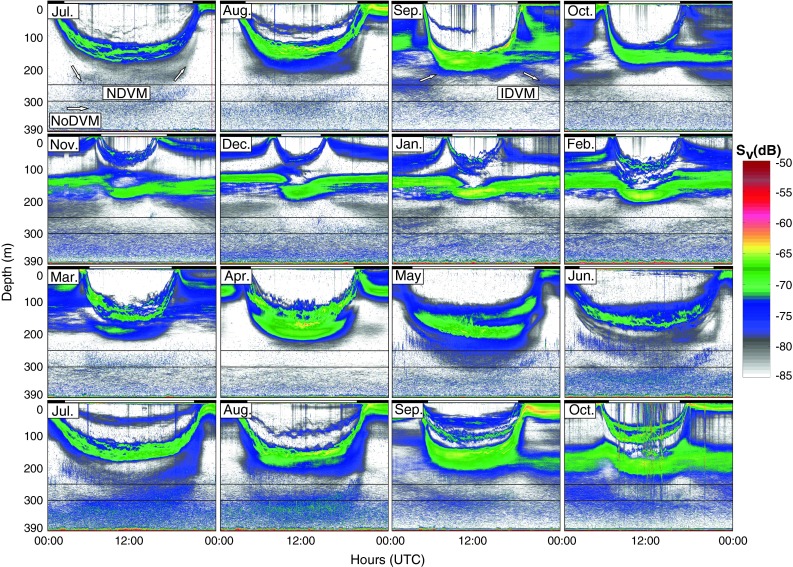
Monthly average echograms from the 38-kHz echo sounder covering the water column (~0–390 m) for 24 h in July 2007–October 2008. The dominant signal (*green*) and backscattering at shallower depth (<200 m) is dominated by pearlside, while glacier lanternfish dominate below ~250 m (see Fig. 3 for clarification). The boxes indicate the intervals where the concentration (individuals m^−3^) of glacier lanternfish is calculated (see Fig. 7). *Arrows* in selected echograms indicate the different behavioral patterns. The *color scale* refers to average volume backscattering (*S*_v_) for each month; *dark red* illustrates the strongest, and *white* the weakest backscatter. The *S*_v_-thresholds are set to −50 and −85 dB for the best possible illustration of the SL’s. *Black* and *white* *bars* above echograms depict night and day separated by times for sunrise and sunset for the 15th each month. Time is given as UTC (local standard time −1 h)

**Fig. 3:**
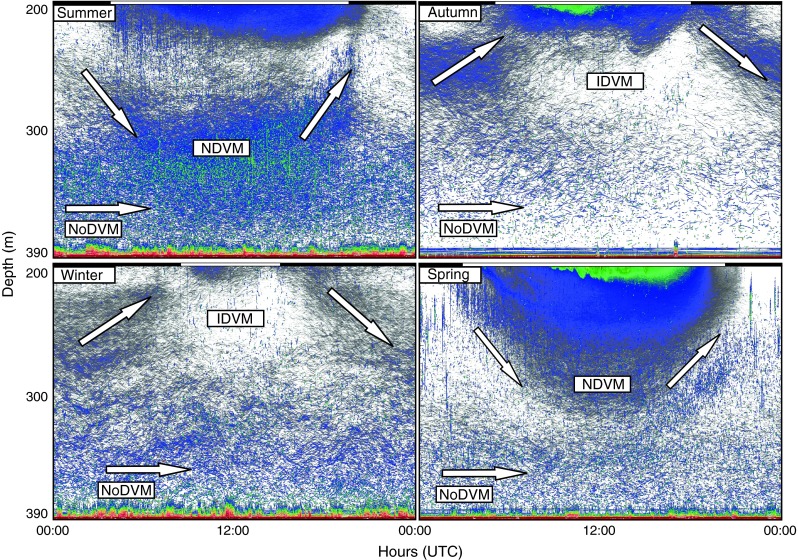
Monthly average echograms from the 38-kHz echo sounder (covering 24 h) illustrating the deeper part of the water column (~200–390 m) in *summer* (represented by August 2008), *autumn* (represented by September 2007), *winter* (represented by January 2008), and *spring* (represented by May 2008). *Arrows* in echograms indicate the different behavioral patterns; No diel vertical migration (*NoDVM*), inverse diel vertical migration (*IDVM*), and normal diel vertical migration (*NDVM*). The *color scale* refers to average volume backscattering (*S*_v_) for each month; *dark red* illustrates the strongest, and *white* the weakest backscatter. *Black* and *white bars* above echograms depict night and day separated by times for sunrise and sunset for the 15th each month. Time is given as UTC (local standard time −1 h)

In summer, there were four SL’s performing NDVM to the surface at night, and one deep layer with no apparent diel behavior (Fig. 2). The upper of these layers (~70–200 m) were ascribed to pearlside as they are found to dominate at these depths (the seasonal DVM patterns of pearlside are discussed in Staby et al. (2011) and will not be addressed in detail here). The two deeper layers (>250 m during daytime) were ascribed to glacier lanternfish with NDVM and NoDVM, respectively. Zooming in on the deeper parts of the water column illustrates more clearly how organisms carrying out NDVM in summer descended to waters below ~250 m at daytime, and ascended at night (Fig. 3).

In autumn, NDVM was still present above ~200 m, but was less noticeable below ~200 m (Fig. 2). IDVM of acoustic targets ascribed to glacier lanternfish was now evident below the pearlside SL (Figs. 2, 3). An apparent non-migrating layer of glacier lanternfish occurred from 300 m to the bottom (Figs. 2, 3).

In winter, there were strong echoes of vertical migrating juvenile pearlside, and largely non-migrating adult pearlside in the upper 200 m (Fig. 2). The SL of individuals with IDVM (glacier lanternfish) was still apparent, although less pronounced than in autumn (Figs. 2, 3). The deepest non-migrating layer was now at its seasonal high in level of backscatter (Fig. 2).

In spring, several SL’s with NDVM appeared between ~100–210 m in daytime (March and April) and in the upper ~100 m at night (Fig. 2). Glacier lanternfish likely contributed in the lower part of this layer. In May, individuals performing NDVM descended to ~300 m in daytime, indicating a large portion of the glacier lanternfish population with this behavior (Figs. 2, 3). As on all other occasions, there was a SL with NoDVM below ~300 m (glacier lanternfish), yet with a seasonal low in backscatter (Fig. 2).

### Relative proportion of migration patterns

**Fig. 4:**
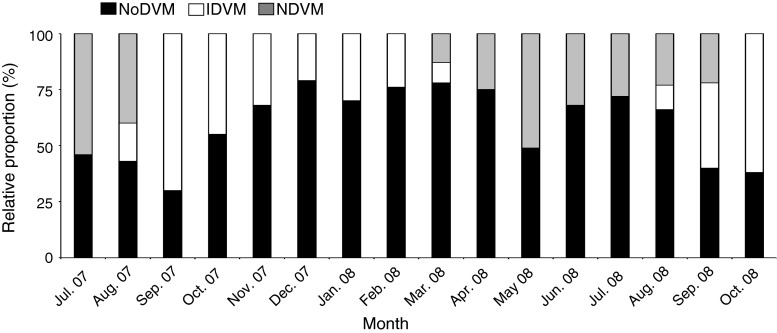
The relative frequency of the population of glacier lanternfish (found below 250 m during daytime) with different DVM behaviors from July 2007–October 2008. No diel vertical migration (*NoDVM*), inverse diel vertical migration (*IDVM*), and normal diel vertical migration (*NDVM*) are illustrated by *black*, *white,* and *gray colors,* respectively

Estimated proportions with different DVM modes were calculated for each month based on the echograms and the echo integration from 250 to 390 m (Fig. 4). NDVM was performed by ~25–55 % of these fish in summer, while this behavior ceased in autumn and winter (Fig. 4). However, individuals performing NDVM re-appeared in early spring, and the proportion performing NDVM increased toward late spring (from ~13 % in March to ~51 % in May) (Fig. 4). The proportion performing IDVM increased from late summer (~15 % in August) to the beginning of autumn (70 % in September 2007), then decreased throughout the winter (24 % in February 2008). In spring and the first part of summer, IDVM was not recorded (Fig. 4). NoDVM was suggested for ~45–75 % of the population at these depths during spring and summer, but this proportion decreased from summer to autumn (~30–40 % in September 2007 and 2008), before it increased toward the winter months (Fig. 4).

### Individual behavior of glacier lanternfish

**Fig. 5:**
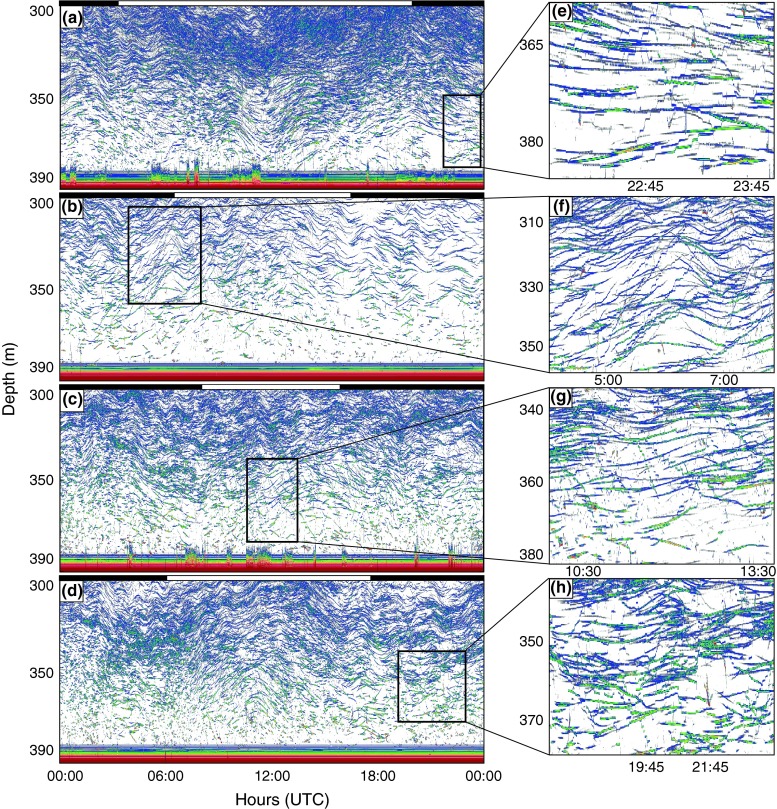
**a**–**d** 24-h Echograms from the bottom-mounted 38 kHz echo sounder on 8 August 2008 (**a**), 18 October 2007 (**b**), 2 February (**c**), and 12 March 2008 (**d**). The echograms are zoomed in at ~300–390 m. The *outlined boxes* are presented in higher resolution in (**e**–**h)**. The *color scale* refers to average volume backscattering (*S*_v_); *dark red* illustrates the strongest, and *white* the weakest backscatter. *Black* and *white bars* above echograms depict night and day separated by times for sunrise and sunset. Time is given as UTC (local standard time −1 h)

Visual inspection of 24 h echograms suggested little systematic vertical swimming among the NoDVM component in relation to time of day, as exemplified by 1 day each season in Fig. 5a–d. Fish at these depths commonly appeared to drift up and down in a consistent pattern suggesting internal wave motions (Fig. 5a–d). The limited vertical movement was reflected in the results from the year-round TT. Of more than 200,000 tracks retrieved by TT, ~85 % revealed vertical relocation speed <0.4 cm/s. However, intermittent vertical swimming was recorded in which individuals ascended or descended in a step-wise pattern (Fig. 5e–h). The maximum vertical swimming speed detected was ~18.4 cm/s. In comparison, the approximately ascending and descending speeds of the migrating layers were 3–4 cm/s for the NDVM layer and 0.5–0.8 cm/s for the IDVM layer. In addition to such marked vertical relocations, seemingly passively drifting fishes once in a while slightly adjusted their vertical distribution, subsequently taking up their apparent torpid behavior.

### Target strength and concentration estimates in deep water

**Fig. 6:**
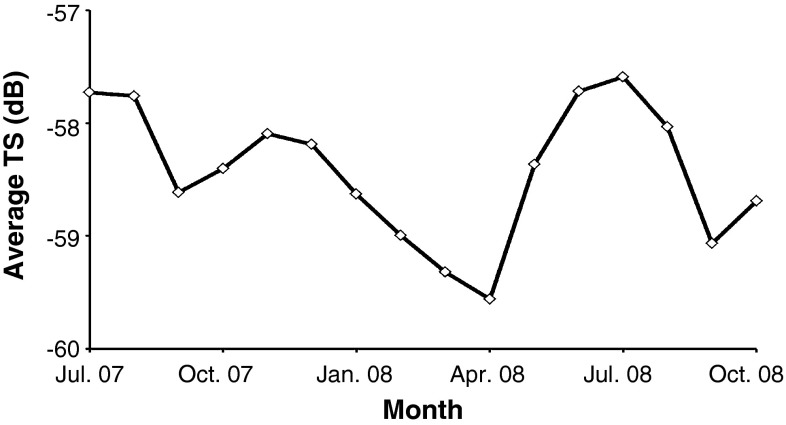
Average TS (dB) of glacier lanternfish (total *n* = 209,768) calculated from ~340 to 380 m for each month throughout the study period (July 2007–October 2008)

The average TS (dB) of glacier lanternfish as measured between ~340 and 380 m were always in the range of approximately −57.5 to −59.5 dB. Within this limited range, the TS distribution of glacier lanternfish showed two peaks (in both summers) and decreased from the autumn to spring (Fig. 6).

**Fig. 7:**
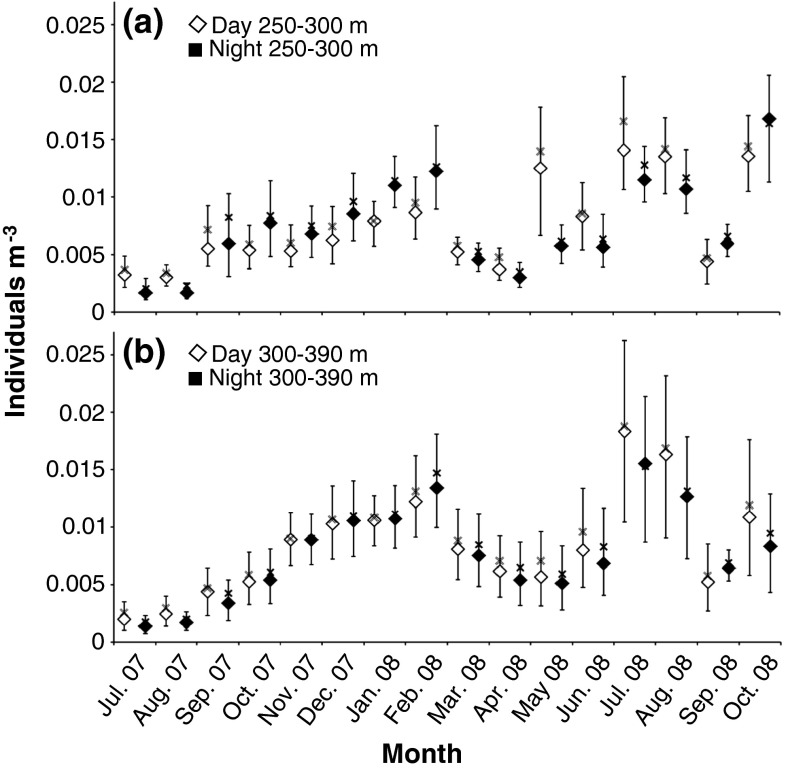
**a**, **b** Estimated monthly average concentration (individuals per m^3^) of glacier lanternfish during day and night from (**a**) 250–300 m and (**b**) 300–390 m during July 2007–October 2008. The *white* and *black squares* indicate the median daytime and nighttime concentration estimates, respectively. The *black lines* illustrate the 1st and 3rd Quartile, while the *X* indicates the average estimated concentration

The TS values were used in assessing the numerical densities of glacier lanternfish. The concentration of fish was usually in the range of 0.005–0.015 individuals m^−3^ (Fig. 7a, b). However, the maximum density, just below 0.02 individuals m^−3^, was recorded in July 2008 in the deepest interval (300–390 m), and in October 2008 in the shallower interval (250–300 m) (Fig. 7a, b).

In spring and summer, when NDVM is the dominating mode of migration, the daytime concentration of the interval ~250–300 m was higher than during night (Fig. 7a). Below 300 m, where the NoDVM mode were dominating, relatively little concentration fluctuations between day and night occurred, except for June–August 2008 (Fig. 7b) when the NDVM layers entered those depths at daytime (Fig. 2). In the months where IDVM were the dominating mode of migration (i.e. September–February), the concentration of glacier lanternfish decreased during daytime in the interval ~250–300 m (Fig. 7a), as the inverse vertical migrators that were distributed from ~220 to 300 m during nighttime ascended to mid-waters (in the lower part of the strong SL between ~150–220 m) during the day (Figs. 2, 3).

## Discussion

The 16-month acoustic data series revealed three different diel behavioral modes of glacier lanternfish. In NDVM, individuals ascend toward the surface at night and descend back to deeper waters in the morning. In IDVM, individuals ascend toward mid-waters at daytime, before descending at night. In NoDVM, individuals stay deeper than ~300 m all day. A clear seasonal pattern, with NDVM prevailing during spring and summer, and IDVM prevailing during autumn and winter, was also apparent. All through the year, NoDVM occurred in the water column deeper than ~300 m. This diversity in diel behavior of glacier lanternfish has previously been observed during autumn in Masfjorden by Kaartvedt et al. (2009), Dypvik et al. (2012), but this is the first assessment of the yearly cycle. Note, however, that while the acoustic results unveil the main patterns in diel behavior for the part of the population below ~200 m, they do not provide the entire picture for the whole population. Only IDVM was recorded acoustically in October and November, yet trawl catches documented some NDVM (Kaartvedt et al. 2009; Dypvik et al. 2012). Also previous studies have documented NDVM of glacier lanternfish in fall and winter (Kaartvedt et al. 1988; Giske et al. 1990; Bagøien et al. 2001). Some shallower-living glacier lanternfish intermingle with, and thus become inseparable in the acoustic signatures of the SL dominated by adult pearlside (Torgersen and Kaartvedt 2001). Shallower-living glacier lanternfish are mostly smaller (Dypvik et al. 2012) and might be too small to be properly detected at 38 kHz. The largely consistency in TS values in deep water underlines that we likely have addressed the adult population throughout this study period. The slight apparent seasonal variation in TS does not seem to correlate with behavioral changes.

During spring and summer, glacier lanternfish performed NDVM toward the surface at night. During this time of year, the concentration of zooplankton peaks in the surface layers, and significantly lowers in the deeper waters (Atkinson and Peck 1988; Rasmussen and Giske 1994; Richter 1994). NDVM among lanternfishes is usually related to avoidance of visual predators in bright surface waters at daytime and subsequent feeding on zooplankton in the surface layers at night (Holton 1969; Dalpadado and Gjøsæter 1987; Kinzer et al. 1993). The pay-off from NDVM will decrease as surface plankton becomes less abundant throughout fall and winter, while at the same time, seasonally migrating copepods return to depth (Speirs et al. 2006; Broms and Melle 2007). This expectedly makes NDVM behavior less profitable. However, small individuals with less energy reserves, larger motivation, or less capability for feeding in deep water may continue to migrate toward the surface at night (Giske and Aksnes 1992), while larger fish may opt for other strategies such as staying in deeper waters feeding on overwintering calanoid copepods. In accordance with this, mainly 2-year or older glacier lanternfish are distributed deeper than ~250 m, while smaller individuals are distributed in shallower waters, during autumn (Dypvik et al. 2012). Similar size-dependent depth distribution has also been observed in other studies of lanternfishes (Willis and Pearcy 1980; Gartner et al. 1987; Auster et al. 1992).

IDVM became apparent in August and was recorded until March. In winter, the bulk of zooplankton biomass is distributed in mid-waters (Giske et al. 1990; Baliño and Aksnes 1993; Bagøien et al. 2001). Dypvik et al. (2012) showed that inversely migrating glacier lanternfish were foraging on overwintering *Calanus* in mid-waters during the day. Other studies have shown that *Calanus* is an important part of the glacier lanternfish diet during fall and winter (Gjøsæter 1973; Baliño and Aksnes 1993). The seasonal occurrence of IDVM seems to harmonize well with the overwintering period for *Calanus finmarchicus* (Bagøien et al. 2001; Speirs et al. 2006), in both the timing of initiation of this behavior, and its termination in spring (Heath 1999; Astthorsson and Gislason 2003; Speirs et al. 2006; Broms and Melle 2007). By staying in deeper waters performing IDVM, glacier lanternfish may optimize their energy budget and reduce the predation risk (Dypvik et al. 2012). In addition, light decreases rapidly with depth in seawater (Sørnes and Aksnes 2006; Aksnes et al. 2009). A lanternfish swimming toward shallower waters, while still staying below ~200 m, will thus experience an increase of light in the surroundings, which would favor visual feeding by the low-light adapted lanternfish (Warrant and Locket 2004). Thus, it seems likely that individuals with IDVM ascend toward the overwintering layer of *Calanus* at daytime to feed at favored light levels, sinking back to deeper layers at night. However, the proportion of the population with IDVM decreases through the winter, which may reflect the reduced concentration of *Calanus* from autumn through winter (Bagøien et al. 2001).

Individuals of glacier lanternfish with NoDVM occurred all year at depths greater than ~300 m. Dypvik et al. (2012) found that the individuals with NoDVM during autumn were the largest individuals of the glacier lanternfish population. Individuals of greatest size tend to have larger eyes and might be better adapted than smaller individuals to detect prey in low light (Warrant and Locket 2004). They are also better adapted for handling large prey such as krill and shrimps, which occur deep in the water column (Kaartvedt et al. 1988; Baliño and Aksnes 1993; Kaartvedt et al. 2009; Dypvik et al. 2012), and which represent a much higher dividend than copepods due to their much larger size (Falk-Petersen 1981; Tande 1982). We therefore suggest that individuals with NoDVM are able to feed in the restricted light levels of the deep waters.

The behavior of individuals in deep water (>300 m) suggested that the NoDVM group indeed largely consisted of non-migrating individuals, and that consistent backscatter in deep water is not a result of asynchronous migrations, but that the population rather splits into migrating and non-migrating individuals, as initially suggested by Pearcy et al. (1979). Nevertheless, single individuals were observed swimming upwards or downwards in a step-wise fashion, as also observed by Kaartvedt et al. (2008, 2009). Intermittent vertical relocations, ascent/descent in a step-wise fashion, would help the fish scan different parts of the water column for prey. Fish may also use their lateral lines for tactile prey detection in darkness (Janssen et al. 1999), and such behavior would be in accordance with both visual (O’Brien et al. 1990) and tactile (Janssen 1997; Janssen et al. 1999; Ryer and Olla 1999) search for prey. There was no evident relation with vertical swimming to time of day. However, the example from October (Fig. 5b) suggests a prominence of ascent at day, so we cannot reject the possibility that some of these deep-living individuals took part in IDVM at that time. Yet, most step-wise movements seemed to represent infrequent re-locations within the layer.

A wide variety of lanternfishes are capable of feeding at depths greater than several hundreds of meters (Pearcy et al. 1979; Roe and Badcock 1984; Sameoto 1988), and NoDVM has previously been observed among several species of lanternfish (Pearcy et al. 1979; Gartner et al. 1987; Collins et al. 2008). IDVM among lanternfishes has not been recorded from other localities. However, different species of *Calanus* occur in different geographic regions and, as well as other genera, perform seasonal vertical migration, overwintering in deeper water (Atkinson and Peck 1988; Richter 1995; Peterson 1998; Broms and Melle 2007). We therefore hypothesize that IDVM and NoDVM are widespread behaviors in areas where concentrations of potential prey are distributed in deeper waters, and that similar plasticity in DVM behavior as observed for glacier lanternfish in Masfjorden might occur in other populations of lanternfishes in areas with seasonally migrating zooplankton, such as overwintering calanoid copepods. Long-term acoustic measurements might be used to reveal such DVM plasticity in other areas, information that could be used to gain knowledge about interactions between lanternfishes, their prey and predators, as well as lanternfishes contribution to the vertical flux of organic matter. The vertical distribution and diel vertical migration of lanternfishes can affect such interactions and processes, making it imperative to further reveal these patterns.
